# The miRNA biogenesis pathway prevents inappropriate expression of injury response genes in developing and adult Schwann cells

**DOI:** 10.1002/glia.23516

**Published:** 2018-10-08

**Authors:** Deniz Gökbuget, Jorge A. Pereira, Lennart Opitz, Dominik Christe, Tobias Kessler, Antonin Marchais, Ueli Suter

**Affiliations:** ^1^ ETH Zurich, Department of Biology Institute of Molecular Health Sciences Zurich Switzerland; ^2^ ETH Zurich/University of Zurich Functional Genomics Center Zurich Zurich Switzerland; ^3^ ETH Zurich, Department of Biology Institute of Agricultural Sciences Zurich Switzerland

**Keywords:** development, injury, miRNAs, peripheral nervous system, Schwann cells

## Abstract

Proper function of the nervous system depends on myelination. In peripheral nerves, Schwann cells (SCs) myelinate axons and the miRNA biogenesis pathway is required for developmental myelination and myelin maintenance. However, regulatory roles of this pathway at different stages of myelination are only partially understood. We addressed the requirement of the core miRNA biogenesis pathway components Dgcr8, Drosha, and Dicer in developing and adult SCs using mouse mutants with a comparative genetics and transcriptomics approach. We found that the microprocessor components Dgcr8 and Drosha are crucial for axonal radial sorting and to establish correct SC numbers upon myelination. Transcriptome analyses revealed a requirement of the microprocessor to prevent aberrantly increased expression of injury‐response genes. Those genes are predicted targets of abundant miRNAs in sciatic nerves (SNs) during developmental myelination. In agreement, Dgcr8 and Dicer are required for proper maintenance of the myelinated SC state, where abundant miRNAs in adult SNs are predicted to target injury‐response genes. We conclude that the miRNA biogenesis pathway in SCs is crucial for preventing inappropriate activity of injury‐response genes in developing and adult SCs.

## 1 INTRODUCTION

A system is defined as robust when it maintains its function in the presence of internal or external perturbations (Kitano, 2004). Posttranscriptional regulation by microRNAs (miRNAs) is an important mean to confer such robustness to developmental transitions and cellular identities (Ebert & Sharp, 2012). miRNAs are a prominent class of small noncoding RNAs that mainly serve to silence their target mRNAs. Biogenesis of most miRNAs requires the sequential processing of the primary miRNA transcript by RNAse III‐like enzymes Drosha, which together with Dgcr8 constitutes the microprocessor, and Dicer to yield a ~22 nucleotide duplex. One strand of the mature miRNA duplex is loaded into Argonaute (Ago) proteins and serves as a guide by imperfect base pairing with target mRNAs, allowing Ago‐dependent recruitment of additional factors and resulting in translational repression, mRNA deadenylation, and mRNA decay. Besides this canonical miRNA biogenesis, some miRNAs (referred to as noncanonical miRNAs) bypass the requirement of the microprocessor or Dicer by being substrates of alternative processing factors such as the spliceosome or the cleaving activity of Ago2 (Ha & Kim, 2014).

Myelination is a crucial feature of the nervous system, fostering refined propagation of electrical signals and providing support to axons. In the peripheral nervous system (PNS), myelination is accomplished by Schwann cells (SCs). These specialized glial cells are derived from the neural crest as the product of differentiating SC precursors (Jessen & Mirsky, 2005; Pereira, Lebrun‐Julien, & Suter, 2012). Initially, immature SCs surround large bundles of axons in groups, while gradually expanding their population as they engage and sort out single axonal segments from the bundles, a process termed radial sorting (Feltri, Poitelon, & Previtali, 2016). After radial sorting is accomplished, myelination is started by SCs that exit the cell cycle and activate a gene program associated with the myelinated SC fate. The resulting myelinating SC state needs to be actively maintained to counteract demyelination‐inducing gene programs and to balance myelin turnover (Decker et al., 2006). In response to peripheral nerve injury, however, myelinating SCs have the remarkable capability to be reprogrammed to repair SCs (also known as Bungner cells) that promote regeneration of injured nerves (Jessen, Mirsky, & Lloyd, 2015).

We have recently found that let‐7 miRNAs drive the onset of myelination (Gökbuget et al., 2015), and loss of Dgcr8 or Dicer in SCs impaired this developmental process (Bremer et al., 2010; Lin, Oksuz, Hurley, Wrabetz, & Awatramani, 2015; Pereira et al., 2010; Verrier, Semple‐Rowland, Madorsky, Papin, & Notterpek, 2010; Yun et al., 2010). Dgcr8‐ablated SCs appeared to be more affected than Dicer‐ablated SCs, in line with potential additional functions of the microprocessor in myelination (Lin et al., 2015). Furthermore, aberrant expression of repair SC markers and failure of proper myelin maintenance were noted. In an injury setting, SC‐specific ablation of Dicer, followed by sciatic nerve (SN) crush, resulted in mildly affected remyelination (Viader, Chang, Fahrner, Nagarajan, & Milbrandt, 2011).

In this study, we aimed to complement the existing knowledge and to gain further insights into the mechanistic functions of the miRNA biogenesis pathway in developmental myelination and myelin maintenance. To achieve this goal, we used a comparative genetic approach to quantitatively dissect the relative requirements and contributions of the SC‐expressed microprocessor‐components Dgcr8 and Drosha, as well as Dicer in regulating the myelinating SC fate. We found that the microprocessor is required for the early onset of radial sorting by promoting the establishment of correct SC numbers. Furthermore, comparative RNA sequencing analysis revealed a regulatory requirement of the microprocessor to prevent inappropriately elevated or de novo expression of injury‐induced genes in developing nerves. Additionally, we found that both Dicer and Dgcr8 are required for proper maintenance of myelinating SCs.

## 2 MATERIALS AND METHODS

### 2.1 Animal models

Mice (*Mus musculus*) depleted of DGCR8, Dicer, or Drosha specifically in SCs were obtained by crossing homozygous *Dgcr8* ^flox/flox^ (Yi et al., 2009), *Dicer1* ^flox/flox^ (Murchison, Partridge, Tam, Cheloufi, & Hannon, 2005), or *Drosha* ^flox/flox^ (Chong, Rasmussen, Rudensky, & Littman, 2008) with *Dhh* ^*Cre*^ mice (Jaegle et al., 2003) heterozygous for *Dgcr8* ^flox/wt^, *Dicer1* ^flox/wt^, or *Drosha* ^flox/wt^. SC‐specific Tamoxifen‐inducible floxed DGCR8 and Dicer mice and Cre‐negative controls were generated by crossing *Dgcr8* ^flox/flox^ or *Dicer1* ^flox/flox^ with *P0‐Cx32* ^*CreERT2*^ mice (Leone et al., 2003) homozygous for *Dgcr8* ^flox/flox^ or *Dicer1* ^flox/flox^. For inducible recombination, 100 μl Tamoxifen (Sigma) (20 mg/ml in sunflower oil, Sigma) was injected *i.p.* on 5 consecutive days in mutant and control animals aged between 8 and 10 weeks. *Dgcr8* ^flox/flox^, *Dicer1* ^flox/flox^, and *Drosha* ^flox/flox^ mice were backcrossed for at least 6 generations, *P0‐Cx32* ^*CreERT2*^ and *Dhh* ^*Cre*^ mice for more than 10 generations, to C57BL/6J. Mice of either gender were used in the experiments, unless indicated otherwise. Transgenic animals were identified by genomic PCR. Age of the analyzed mice is specified in the respective figure legends. The veterinary office of the Canton of Zurich approved all animal experiments in this study.

### 2.2 Reagents

For a detailed list of reagents used in this study, see Supporting Information, Table S1.

### 2.3 miRNA target prediction

miRNA target prediction was based on TargetScan for mouse. We used the R/Bioconductor package targetscan.Mm.eg.db (version 0.6.1) to extract predicted targets from TargetScan (Supporting Information, Files S1 and S2).

### 2.4 Crush injury surgery

Two‐month‐old C57BL/6J male mice were injected with buprenorphinum (Temgesic, Reckitt Benckiser) at a dosage of 0.1 mg/kg of bodyweight. The first injection was prior to the surgery, and treatment was maintained up to 2 days postsurgery. The surgery was performed under isoflurane anesthesia. An incision was performed at the height of the hip, and the sciatic nerve exposed on one side. The nerve was crushed using Dumont S&T JF‐5 forceps (FST tools) by compressing the same site (at the sciatic notch) 3 times in a row for 10 s each. The wound was sealed, and the animals euthanized 3 days after the surgery. A 7 mm piece of the injured sciatic nerve was harvested from 1 mm up to 8 mm distal to the injury site. The contralateral noninjured sciatic nerve was also harvested and processed in parallel.

### 2.5 Electron microscopy

Animals were euthanized by injection of Pentobarbital (150 mg/kg *i.p.*). Subsequently, harvested tissue was fixed with 3% glutardialdehyde (Sigma) and 4% paraformaldehyde (Electron Microscopy Sciences—EMS) in 0.1 M phosphate buffer. Next, the tissue was treated with 2% osmium tetroxide (EMS), dehydrated over a series of acetone (Merck) gradients and embedded in Spurrs resin (EMS). Imaging of ultrathin sections (65 nm) was performed using a FEI Morgagni 268 TEM for high‐resolution micrographs. For morphological quantifications, additional sections (99 nm) were harvested. These sections were collected on ITO coverslips (Optics Balzers) and the complete surface of the nerve section was imaged and analyzed using a Zeiss Gemini Leo 1530 FEG or Zeiss Merlin FEG scanning electron microscopes attached to ATLAS modules (Zeiss), allowing for imaging of large areas at high resolution.

### 2.6 RNA sequencing

Small RNA sequencing data from sciatic nerves at P1 and P60 were retrieved from the dataset available at the NCBI GEO database (GSE64562) (Gökbuget et al., 2015).

For long RNA sequencing, RNA from sciatic nerves of conditional inducible mutant animals (*P0‐Cx32* ^*CreERT2*^ heterozygous, and homozygous for either *Dicer1* ^flox/flox^ or *DGCR8* ^flox/flox^, three mice from each genotype) and their respective controls (negative for Cre, and *Dicer1* ^flox/flox^ or *DGCR8* ^flox/flox^, three mice from each genotype), and conditional mutant animals (*Dhh* ^*Cre*^ *DGCR8* ^flox/flox^ and *Dhh* ^*Cre*^ *Drosha* ^flox/flox^, three mice from each genotype) and Cre‐negative controls (three mice) was extracted with Qiazol (Qiagen) following the manufacturer's instructions. The same way, RNA was extracted from sciatic nerves of 3 dpc C57BL/6J male mice (four mice) and contralateral (four mice) and uncrushed controls (three mice). RNA quality control, library preparation, sequencing, and mapping of reads were performed at the Functional Genomics Center in Zurich (FGCZ). The TruSeq Stranded total RNA Sample Prep Kit (Illumina, Inc, CA) in combination with ribosomal RNA removal with mouse‐specific probes (RiboZero‐Gold) was used to produce RNA‐seq libraries. The quality and quantity of the generated libraries were determined by Agilent Technologies 2100 Bioanalyzer with DNA‐specific chip and quantitative PCR (qPCR) using Illumina adapter‐specific primers using the Roche LightCycler system (Roche Diagnostics). The libraries were sequenced on HiSeq2500 in paired end read mode at 2× 125 bp. NGS reads were quality‐checked with FastQC (http://www.bioinformatics.babraham.ac.uk/projects/fastqc/). Reads were trimmed with Trimmomatic (Bolger, Lohse, & Usadel, 2014) (version 0.33, 4 bases hard‐trimming from the start, and adapter trimming at the end). We aligned the trimmed reads to the reference genome and transcriptome (FASTA and GTF files, respectively, Ensembl GRCm38) with STAR (Dobin et al., 2013) version 2.5.1b. Gene expression was quantified using RSEM (Li & Dewey, 2011) (version 1.2.22). To detect differentially expressed genes, we applied the count‐based negative binomial model implemented in the R/Bioconductor package edgeR (version 3.12) (Robinson, McCarthy, & Smyth, 2010), in which the normalization factor was calculated by trimmed mean of *M* values (TMM) method (Robinson & Oshlack, 2010). For sample clustering and visualization, we used the software Biolayout Express3D (v3.2, http://www.biolayout.org/). Heatmaps in the respective figures were generated with the R package gplots (v3.0.1) using the heatmap2‐function. To generate Venn plots, we used the R package VennDiagram (v1.6.17). The long RNA sequencing data have been deposited at the NCBI GEO database with the primary accession code GSE109075. The long RNA sequencing data for conditional Dicer mutants and controls was retrieved from the dataset available at the NCBI GEO database (GSE64562) (Gökbuget et al., 2015).

### 2.7 Immunofluorescence, TUNEL, and EdU detection

Tissues were fixed in 4% PFA and embedded in OCT compound (Sakura) after successive incubations in 10% and 20% sucrose (Sigma) in PBS. Cryosections were cut at a thickness of 10 μm and collected on Superfrost Plus slides (Thermo Scientific). For stainings, sections were fixed with 4% PFA for 10 min. For analysis of proliferation, sections were blocked for 20 min with PBS containing 3% bovine serum albumin (BSA, Sigma) and 0.5% Triton‐X100 (Sigma) followed by two washes in 3% BSA in PBS. Detection of EdU incorporation was performed by a 30 min incubation of each slide with PBS containing 20 mM CuSO_4_, 4 mM Sodium‐(+)‐l‐ascorbate and 2 μM Alexa Fluor 647 Azide (Thermo Scientific). For TdT‐mediated dUTP‐biotin nick end labeling (TUNEL), slides were blocked 1 hr in PBS containing 5% normal goat serum NGS (Life Technologies), 0.1% BSA, and 1% Triton‐X‐100 (Sigma). Labeling and detection was performed according to manufacturer's instructions (Roche). For cleaved‐Caspase 3 stainings, slides were incubated successively in 0.5% Triton‐X‐100 in PBS for 20 min and in PBS containing 5% donkey serum (Millipore), 0.5% BSA, and 0.1% Triton‐X‐100 for 30 min. Primary antibodies were rabbit‐anti‐Cleaved‐Caspase‐3 (9661, Cell Signaling, 1:400), goat‐anti‐Sox10 (AF2864, R&D, 1:100), and rat‐anti‐CD68 (MCA1957, Serotec, 1:200). Alexa‐dye conjugated secondary antibodies were used at a dilution of 1:500. Nuclei were stained by incubation with 0.1 ng/ml DAPI (Sigma) in PBS for 10 min. Microscopy was performed with the Axio Imager.M2 (Zeiss). Images were quantified using Adobe Photoshop CS5 or CS6 on at least two sections, or more than 450 cells per animal.

### 2.8 Reverse transcription and real‐time PCR

Frozen SNs were freed of peri‐ and epineurial layers and homogenized in 20 μl Qiazol (Qiagen) using chilled pestles. After addition of another 480 μl of Qiazol, total RNA was extracted according to the Qiazol manufacturer's instructions (Qiagen). RNA precipitation was enhanced using GlycoBlue Coprecipitant (Thermo Scientific). Taqman miRNA assays were performed according to manufacturer's instructions using 2–5 ng total RNA input (Applied Biosystems). Reverse transcription of total RNA was performed using 50–100 ng input with Maxima First Strand cDNA Synthesis Kit (Thermo). Subsequent quantitative real‐time PCR was performed with 2× FastStart Essential DNA Green Master Mix (Roche) on a Lightcycler 480II (Roche) using 2 μl 1:10 diluted total cDNA. PCR reactions were carried out with a minimum of three technical replicates per sciatic nerve sample. Primer sequences are supplied in Supporting Information, Table S2.

### 2.9 Gene set enrichment analysis

Gene set enrichment analysis was performed using the “GO Biological Process 2015” and “KEGG 2016” gene sets embedded in the Enrichr tool (Chen et al., 2013). Output lists (Supporting Information, Files S3–S7) were converted into the “Generic results” file format of the Enrichment Map Tool for Cytoscape 3 (Merico, Isserlin, Stueker, Emili, & Bader, 2010), which was used with an FDR cutoff 0.05. Summarized cluster names were computed with the WordCloud Cytoscape 3 plugin (Oesper, Merico, Isserlin, & Bader, 2011).

### 2.10 Statistical analysis

Statistics were performed using GraphPad Prism 6. Statistical significance of two‐group comparisons was assessed with the two‐sided two‐sample Student's *t* test and of multiple group comparisons with the two‐way anova with Tukey's multiple comparisons test was used, unless stated otherwise. Data distribution was assumed normal and variances were assumed equal, although these were not formally tested due to low sample sizes. Detailed information about error bars and sample sizes are included in all figure legends with further specifications in the Experimental Procedures section (see above). Sample size was not predetermined by statistical methods. Randomization was not applied unless stated otherwise. No blinding was applied with exception to all morphometric quantifications of electron micrographs, which were performed blinded. No samples or data were excluded from the analysis.

## 3 RESULTS

### 3.1 Axonal radial sorting requires microprocessor function in Schwann cells

**Figure 1:**
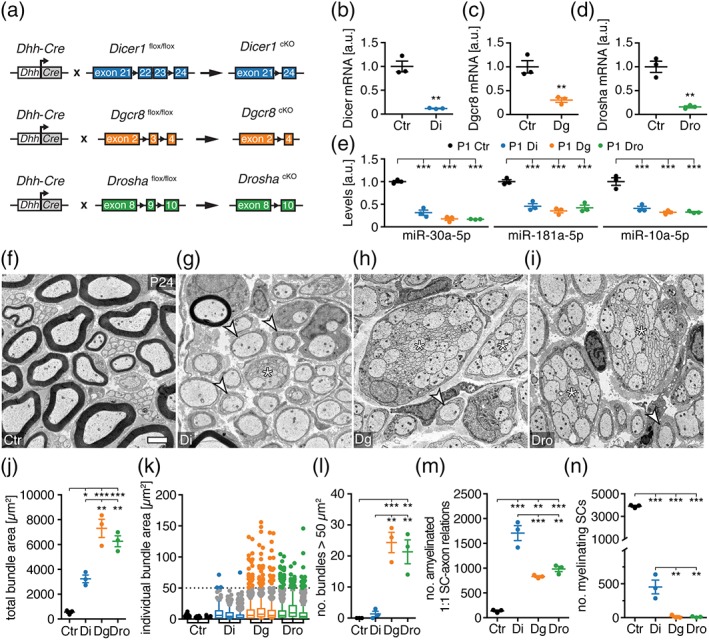
The microprocessor is required for radial sorting. (a) Scheme of conditional SC‐specific Dicer, Dgcr8, and Drosha deletion strategy. (b–d) mRNA levels of Dicer in *Dhh* ^*Cre*^ *‐Dicer1* ^*flox/flox*^ (Di) (b), Dgcr8 in *Dhh* ^*Cre*^ *‐Dgcr8* ^*flox/flox*^ (Dg) (c), and Drosha in *Dhh* ^*Cre*^ *‐Drosha* ^*flox/flox*^ (Dro) (d) compared to controls (Ctr) normalized to β‐actin mRNA in P1 SN (*n* = 3 mice per condition). (e) Levels of miR‐30a‐5p, miR‐181a‐5p, and miR‐10a‐5p in Di, Dg, and Dro compared to Ctr, normalized to sno‐202 in P1 SN (*n* = 3 mice per condition). (f–i) Electron micrographs of SNs of Ctr, Di, Dg, and Dro at P24. Examples of 1:1 SC‐axon amyelinated units (white arrowheads) and bundles of unsorted axons are highlighted (asterisks). (j) Total area covered by bundles of unsorted axons per SN cross‐section in Ctr, Di, Dg, and Dro at P24 (3 mice per condition). Unsorted bundles in mutants are defined by at least two axons in direct contact with each other, without an SC process in‐between. Bundles in controls are small caliber axons as part of Remak bundles undergoing maturation. (k) Distribution of individual bundle sizes per SN cross‐section of Ctr, Di, Dg, and Dro at P24 (one box plot per animal). Bundles larger than 50 μm^2^ are highlighted as colored data points. (l) Number of bundles of unsorted axons covering an area larger than 50 μm^2^ in SNs of Ctr, Di, Dg, and Dro at P24 (3 mice per condition). (m,n) Number of 1:1 SC‐axon amyelinated units (m) and myelinating (n) SCs per SN cross‐section in Ctr, Di, Dg, and Dro at P24 (3 mice per condition). Scale bar, 2 μm (f–i). Error bars: *SEM.* (b–e,j,l–n). Two‐sided two‐sample Student's *t* test (b–d) or one‐way anova with Tukey's multiple comparison test (e,j,l–n). *p < .05, **p < .01, ***p < .001 [Color figure can be viewed at wileyonlinelibrary.com]

To assess similarities and differences in the requirement of miRNA biogenesis pathway components Drosha, Dgcr8, and Dicer in developing SCs quantitatively and in parallel, we generated conditional SC‐specific loss‐of‐function mice (*Mus musculus*) for Dicer (Dicer cKO), Dgcr8 (Dgcr8 cKO), and Drosha (Drosha cKO) using a previously established Cre recombinase system expressed under *Dhh* gene regulatory sequences (Figure 1a) (Jaegle et al., 2003). As expected, the levels of Dicer, Dgcr8, and Drosha mRNAs and SN‐enriched miRNAs (Gökbuget et al., 2015) were strongly diminished in respective mutant mice at postnatal day (P) 1 (Figure 1b–e). Morphological analysis of SNs at P24 revealed numerous aberrant large bundles of unsorted axons, usually a unique feature of SC differentiation during the early stage of radial sorting, specifically in Dgcr8 cKO and Drosha cKO (Figure 1f–i). SNs of Dicer cKO were mainly arrested at the promyelinating (i.e., sorted but unmyelinated 1:1 SC‐axon) stage as previously described (Bremer et al., 2010; Pereira et al., 2010; Verrier et al., 2010; Yun et al., 2010), with a few small bundles remaining (Figure 1g). Quantifications confirmed an increased total SN area occupied by bundles and increased numbers of large bundles in Dgcr8 and Drosha cKO compared to Dicer cKO (Figure 1j–l). In addition, the number of SC‐axon units arrested at the promyelinating stage was decreased in Dgcr8 and Drosha cKO compared to Dicer cKO, and myelinated axons were almost completely absent (Figure 1m,n). These data indicate a specific developmental impairment of Dgcr8 and Drosha cKO. Notably, the finding is not dependent on a potential detrimental role of unsuppressed Dicer activity if the microprocessor is inactive, as simultaneous SC‐specific deletion of both Dicer and Dgcr8 (Dicer–Dgcr8 dKO) morphologically mimics the Dgcr8 and Drosha cKO phenotypes (Supporting Information, Figure S1). Furthermore, a confounding role of putative differences in genetic background is unlikely based on examinations of crossbred Dgcr8 and Dicer lines for multiple generations. Homozygous Dgcr8 mutants (heterozygous for Dicer) generated from these matings reproduced the developmental impairment displayed by Dgcr8 cKOs. Littermate homozygous Dicer mutants (heterozygous for DGCR8) closely resembled Dicer cKO (Supporting Information, Figure S1). Interestingly, no mature Remak bundles were found in any of the mutants mentioned above. Taken together, ablation of Dgcr8 and Drosha in SCs revealed a critical requirement of the microprocessor for early onset radial sorting, a necessity which is less evident when SCs lack Dicer.

### 3.2 The microprocessor ensures accurate numbers of Schwann cells during radial sorting

**Figure 2:**
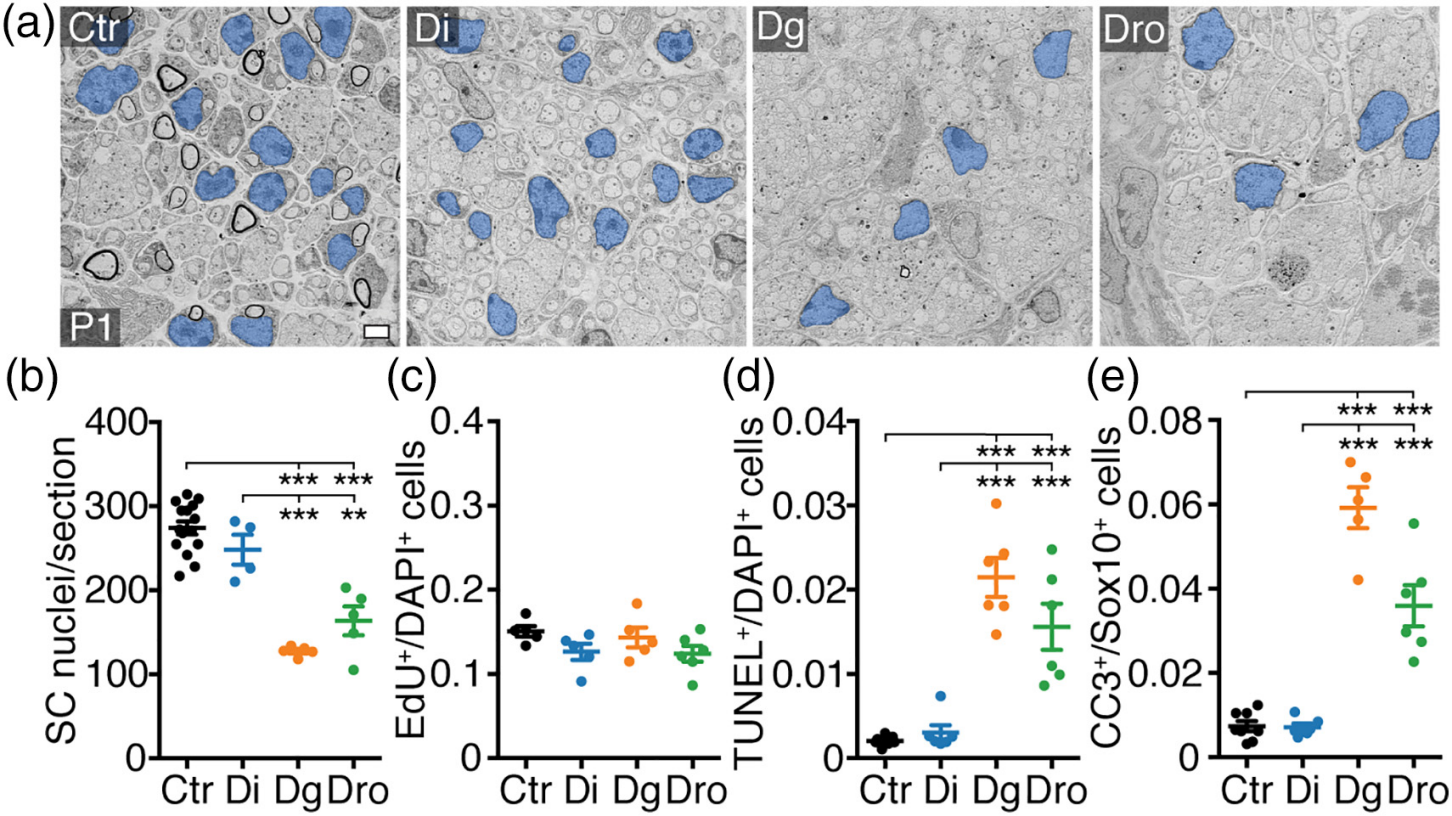
The microprocessor is required to establish correct SC numbers during radial sorting. (a) Electron micrographs of SNs of controls (Ctr), *Dhh* ^*Cre*^ *‐Dicer1* ^*flox/flox*^ (Di), *Dhh* ^*Cre*^ *‐Dgcr8* ^*flox/flox*^ (Dg), and *Dhh* ^*Cre*^ *‐Drosha* ^*flox/flox*^ (Dro) at P1. SC nuclei are false colored in blue. (b) Number of SC nuclei per SN cross‐section in Ctr, Di, Dg, and Dro at P1. Number (*n*) of animals per condition: Ctr (*n* = 15), Di (*n* = 4), Dg (*n* = 5), Dro (*n* = 5). Ctr are also represented in Supporting Information, Figure S2a. (c) Number of EdU‐positive per DAPI‐positive cells in SNs of Ctr, Di, Dg, and Dro at P1. Number (*n*) of animals per condition: Ctr (*n* = 5), Di (*n* = 5), Dg (*n* = 5), and Dro (*n* = 6). (d) Number of TUNEL‐positive per DAPI‐positive cells in SNs of Ctr, Di, Dg, and Dro at P1. Number (*n*) of animals per condition: Ctr (*n* = 7), Di (*n* = 6), Dg (*n* = 6), and Dro (*n* = 6). (e) Number of cleaved caspase 3 (CC3)‐positive per Sox10‐positive SCs in SNs of Ctr, Di, Dg, and Dro at P1. Number (*n*) of animals per condition: Ctr (*n* = 8), Di (*n* = 6), Dg (*n* = 5), and Dro (*n* = 6). *p*(Dg vs Dro) = .0004. Scale bar, 2 μm (a). Error bars: *SEM.* One‐way anova with Tukey's multiple comparison test *p < .05, **p < .01, ***p < .001 (b–e) [Color figure can be viewed at wileyonlinelibrary.com]

Next, we addressed the cellular mechanism connected to the pronounced early onset radial sorting defect observed upon microprocessor deletion. Successful radial sorting depends on, among other factors, correct expansion of the SC population to match the number of available axonal segments to be myelinated (Feltri et al., 2016). Failure to do so results in early onset radial sorting defects as observed in Dgcr8 and Drosha cKO. To address whether there are sufficient SCs in these mutants, we quantified the number of SCs in SNs of Dicer cKO, Dgcr8 cKO, Drosha cKO, and controls. We found fewer SCs in Dgcr8 and Drosha cKO (P1) compared to Dicer cKO and controls (Figure 2a,b and Supporting Information, Figure S2a,b). Further examinations revealed no detectable differences of EdU incorporation in SNs of Dicer, Dgcr8, and Drosha cKO compared to controls at P1 (Figure 2c). These findings indicate that the observed reduction in SC numbers is likely not due to reduced proliferation. Analysis of cell death, however, revealed more cleaved‐Caspase‐3 (CC3)‐positive SCs and more TUNEL‐positive cells in SNs of P1 Dgcr8 and Drosha cKO, but not in Dicer cKO, in comparison to controls (Figure 2d,e). Taken together, our data support that the microprocessor is required to ensure the correct SC number upon onset of radial sorting, at least in part by promoting SC survival.

### 3.3 The microprocessor precludes incorrect upregulation of injury‐response genes in developing Schwann cells

**Figure 3:**
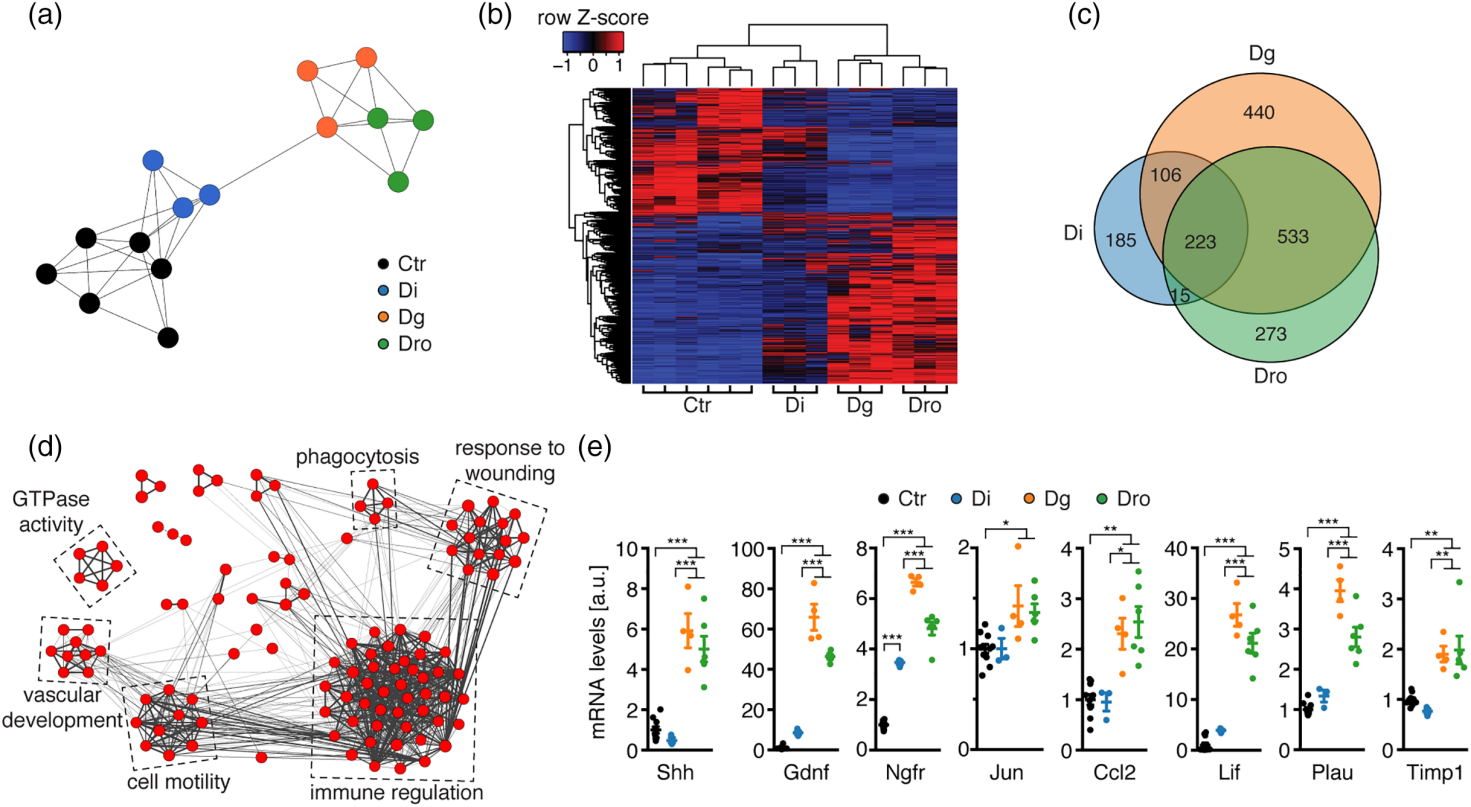
The microprocessor prevents inappropriately upregulated expression of injury‐response genes in developing SCs. (a) Sample‐to‐sample Pearson correlation clustering performed with BioLayout Express3D for log_2_‐transformed FPKM values of each analyzed control (Ctr), Dicer cKO (Di), Dgcr8 cKO (Dg), and Drosha cKO (Dro) mouse at P1. (b) Heatmap of differentially expressed (fold change ≥2, FDR ≤ 0.05) genes in Dg and Dro in comparison to Di and Ctr at P1. Color intensities represent the *Z*‐score for each row, red indicating higher expression and blue indicating lower expression. Columns and rows were organized by unsupervised clustering. (c) Venn diagram comparing upregulated genes (fold change ≥2, FDR ≤ 0.05) in RNA sequencing data from Di, Dg, and Dro compared to Ctr at P1. (d) Network graph output of enrichment map produced by Cytoscape 3 showing GO biological process gene sets (FDR < 0.05) significantly enriched among the 533 upregulated genes in both Dg and Dro. Nodes represent individual GO term gene sets. Edges represent the relatedness between the genes within the gene sets. Immune regulation and injury‐related clusters are prominent. (e) Relative mRNA levels of Shh, Gdnf, Ngfr, Jun, Ccl2, Lif, Plau, and Timp1 in SNs of Ctr, Di, Dg, and Dro at P1. Number (*n*) of animals per condition: Ctr (*n* = 10 for Shh, Gdnf, Ngfr, Ccl2, Plau, and Timp1; *n* = 11 for Jun and Lif), Di (*n* = 3), Dg (*n* = 4), and Dro (*n* = 6). Error bars: *SEM.* One‐way anova with Tukey's multiple comparison test summarized per mRNA species as *p < .05, **p < .01, ***p < .001 [Color figure can be viewed at wileyonlinelibrary.com]

To gain insights into the molecular changes linked to the early onset radial sorting defects observed upon microprocessor ablation, we performed whole‐transcriptome analysis by RNA sequencing of SNs of P1 Dgcr8 and Drosha cKO compared to Dicer cKO and controls. Evaluation of sample‐to‐sample Pearson correlation revealed clustering of Dgcr8 with Drosha cKO and Dicer cKO with controls. These findings indicate an overall similarity of Dgcr8 and Drosha cKO also on the gene expression level and show an overall difference from Dicer cKO and controls (Figure 3a). Hierarchical cluster analysis of differentially expressed transcripts further confirmed the similarities of gene expression profiles of Dgcr8 and Drosha cKO, and the differences towards Dicer cKO and controls (Figure 3b). To determine which biological processes are associated with these differences, we performed a gene ontology (GO) term enrichment analysis using the Enrichr tool (Chen et al., 2013). Given that the microprocessor mainly acts by giving rise to miRNAs that suppress target mRNAs, or in certain settings by directly binding and cleaving target mRNAs, we focused our analysis on the 533 genes robustly upregulated in Dgcr8 and Drosha cKO, qualifying them as potential primary effectors that drive the observed phenotypic differences (Figure 3c). Clustering of significantly enriched gene sets based on their degree of gene overlap using the Enrichment Map network visualization tool (Merico et al., 2010) yielded six major clusters (Figure 3d). Interestingly, multiple clusters are associated with immune system‐ and injury‐related functions, in line with previous findings implicating de novo expression of repair SC markers upon SC‐Dgcr8 ablation (Lin et al., 2015). Using qRT‐PCR on P1 SN lysates, we observed a specific enrichment of repair SC markers, including Shh and Gdnf (Jessen & Mirsky, 2016), and of transcripts that haven been previously associated with the inflammatory (Ccl2/Mcp1, Lif, Timp1, Plau) and the dedifferentiation component (Jun, Ngfr/p75) of the injury response (Arthur‐Farraj et al., 2012; Fontana et al., 2012; Napoli et al., 2012) (Figure 3e) in Dgcr8 and Drosha cKO. The elevated Ccl2 and Lif levels are not due to increased macrophage numbers (Supporting Information, Figure S2c), or a developmental delay (Supporting Information, Figure S2d,e), arguing toward de novo upregulation of these cytokines. Interestingly, miRNA target site prediction revealed conserved seed matches for SN‐enriched miRNAs at P1 (Gökbuget et al., 2015) within the 3′‐UTRs of Lif, Ngfr and Gdnf (Supporting Information, Figure S2f). Of note, the 533 transcripts upregulated in Dgcr8 and Drosha cKO show also a trend toward upregulation in Dicer cKO (Supporting Information, Figure S2g). These findings concur with the interpretation of miRNAs as major regulatory effectors in our mutants. Furthermore, the data raise the distinct possibility that differences in miRNA depletion efficiencies may underlie some of the more robust upregulations found in Dgcr8 and Drosha cKO.

GO analyses of downregulated genes in Dgcr8 and Drosha cKO yielded mainly terms associated with myelination, likely reflecting the observed developmental delay compared to Dicer cKO and controls (Supporting Information, Figure S2h).

In summary, our molecular analysis revealed major differences in gene expression of Dgcr8 and Drosha cKO compared to Dicer cKO and controls, with a particular enrichment for injury‐related gene sets.

### 3.4 Microprocessor‐dependent injury‐response genes are expected targets of abundant miRNAs and differentially expressed transcription factors

Given the de novo appearance or accumulation of repair SC markers and injury‐related transcripts in response to microprocessor ablation in developing SCs, we sought to address to what extent this observed injury response matches the physiological response of SNs after injury. Thus, we performed whole‐transcriptome analysis by RNA sequencing of wild‐type mouse SNs 3 days post crush injury (dpc) in comparison to uncrushed and contralateral control nerves. We found 2802 significantly upregulated (≥twofold) and 1550 significantly downregulated genes (≤0.5‐fold) in 3 dpc SNs compared to uncrushed and contralateral control nerves (Supporting Information, Figure S3a). GO term gene set enrichment analysis of these differentially upregulated genes unveiled large clusters associated with immune system functions such as immune regulation, leukocyte migration, response to injury, lymphocyte activation, blood coagulation, and neutrophil migration (Supporting Information, Figure S3b). Other large clusters were associated with protein localization, cell cycle, nucleosome assembly, telomere maintenance, GTPase activity, and negative regulation of phosphorylation. The significantly downregulated genes were mainly associated with fatty acid biosynthesis and cholesterol biosynthesis (Supporting Information, Figure S3b).

**Figure 4:**
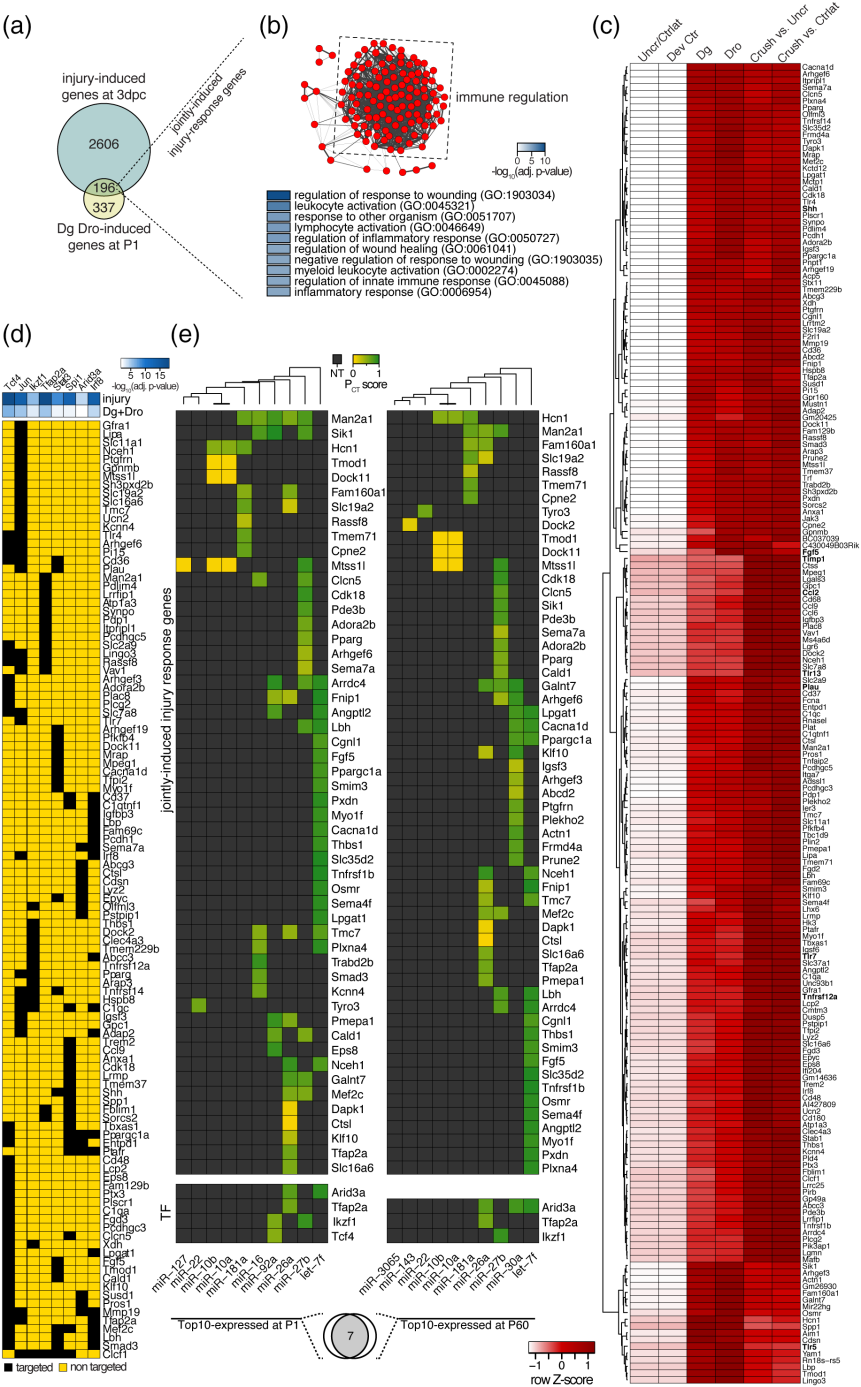
Microprocessor‐dependent injury‐response genes are predicted targets of abundant miRNAs and differentially expressed transcription factors. (a) Venn diagram of injury‐induced genes (3 dpc) and genes induced in Dgcr8 and Drosha cKO. 196 genes are induced in both conditions (termed jointly induced injury‐response genes). (b) Network graph produced by Cytoscape 3 showing significantly enriched GO biological process gene sets among the common injury‐response genes imported into Enrichment Map (top panel). Nodes represent individual GO term gene sets. Edges represent the relatedness between the genes within the gene sets. Genes are highly enriched for immune regulatory and injury‐related functions. Top 10 significantly enriched GO terms are shown in the panel below. (c) Heatmap of jointly expressed injury‐response genes. Color intensities represent row *Z*‐score, red indicating higher expression and white indicating lower expression. (d) Upregulated transcription factors (TFs) with predicted binding motifs in the promoter regions of jointly differentially expressed injury‐response genes (i.e., both upregulated and downregulated) in 3 dpc SNs compared to contralateral and uncrushed controls and in SNs of Dg and Dro at P1 compared to controls (fold change ≥2, FDR ≤ .05). Negative decadic logarithm of adjusted *p* value of TF target enrichment among injury induced genes (injury) and genes induced in Dgcr8 and Drosha cKO (Dg + Dro) is color‐coded (white indicating high and blue low *p* values). Targeted (black) and nontargeted (yellow) jointly differentially expressed injury‐response genes are shown for each TF. (e) Top 10 expressed miRNAs in P1 and P60 SNs of wild‐type mice and their predicted target sites (based on TargetScan) among the jointly induced injury‐response genes and upregulated TFs predicted to target jointly differentially‐expressed injury‐response genes. Target site conservation and quality is represented by the P_CT_ score and nonpredicted targets (NT) in grey. Venn diagram below indicating 7 out 10 most abundant miRNAs overlap between P1 and P60 [Color figure can be viewed at wileyonlinelibrary.com]

To examine which genes are both upregulated in expression upon SN injury and after developmental microprocessor ablation, we compared the differentially upregulated genes in 3 dpc SNs with those upregulated in both, Dgcr8 and Drosha cKO. We found that 196 out of 533 (37%) genes induced in developing Dgcr8 and Drosha cKO were also upregulated upon SN injury designated “jointly induced injury‐response genes” (Figure 4a). These jointly induced injury‐response genes were highly enriched for immune regulatory functions such as the GO terms “regulation of response to wounding,” “leukocyte activation,” “response to other organism,” “lymphocyte activation,” and “regulation of wound healing” (Figure 4b,c). Besides previously described injury‐induced genes such as Ccl2, Shh, Fgf5, Timp1, Plau, and several Toll‐like receptors (Arthur‐Farraj et al., 2012; Bosse, Hasenpusch‐Theil, Kury, & Muller, 2006; Martini, Fischer, Lopez‐Vales, & David, 2008; Napoli et al., 2012), we found also genes with no previously described function during the SN‐injury response such as Tfap2a and Lingo3. The induction of this partial injury‐response gene program in Dgcr8 and Drosha mutants during early development suggests a requirement of the microprocessor to prevent aberrant activity of the SC repair program during normal development.

We next sought to apply an unbiased bioinformatics approach to explore putative regulatory networks underlying the differential expression of injury‐response genes. Given that miRNAs often target central signaling hubs such as transcription factors to shape developmental gene expression (Ebert & Sharp, 2012), we predicted conserved transcription factor‐binding sites within the promoter regions of all jointly differentially expressed injury‐response genes (i.e., both upregulated and downregulated) upon SN injury and in Dgcr8 and Drosha cKO using the TRANSFAC/JASPAR tool embedded within Enrichr. For the genes differentially expressed at 3 dpc, we identified 233 significantly enriched transcription factor motifs. For the differentially expressed genes in Dgcr8 and Drosha cKO, we found 147 significantly enriched transcription factor (TF) motifs. Out of both sets, 145 were common. When we required additionally that motif‐associated TFs had also to be upregulated upon injury and microprocessor ablation, we came up with eight candidates (Tcf4, c‐Jun, Ikzf1, Tfap2a, Stat3, Spi1, Arid3a, and Irf8) that are predicted to regulate jointly differentially expressed injury‐response genes (Figure 4d). The identified TFs included c‐Jun, an already known key regulator of the injury response (Arthur‐Farraj et al., 2012; Fontana et al., 2012) and interestingly other TFs that have not been previously implicated in the nerve injury response, such as Tfap2a, Tcf4, and Irf8 (Figure 4d).

Next, we took advantage of previously reported small RNA sequencing data (Gökbuget et al., 2015) from wild‐type SNs to ask, whether miRNAs that are abundant during SN development and in adulthood have predicted conserved binding sites within transcripts of jointly induced injury‐response genes (Figure 4a) and within transcripts of the eight TFs that are predicted to regulate the jointly differentially expressed injury‐response genes (Figure 4d). We found that the 10 most abundant miRNAs in SNs at P1 and P60 account for 60%–65% of all detected mature miRNA reads (Supporting Information, Figure S3c–e), a value in the range of what is considered as functionally relevant expressed (Olive, Minella, & He, 2015). Seven out of these 10 miRNAs are common between P1 and P60 (let‐7f, miR‐27b, miR‐26a, miR‐181a, miR‐10a, miR‐10b, and miR‐22) (Figure 4e). Out of the 196 jointly induced injury‐response genes (Figure 4a), 52 are predicted targets of the top 10 miRNAs at P1 (Supporting Information, File S1), 57 are predicted targets of the top 10 miRNAs at P60 (Supporting Information, File S2), and 48 are predicted as targets at both ages by this analysis. Out of the eight TFs in question, Tfap2a, Arid3a, and Ikzf1 are also predicted to be targets of P1 and P60‐enriched miRNAs. Taken together, these data predict that abundant developmental stage‐specific miRNAs indeed target a subset of transcripts of jointly induced injury‐response genes (Figure 4a) and by projected upstream TFs. The predictive analyses support the existence of a complex network of miRNAs targeting injury‐induced transcripts, including TFs that regulate the expression of such affected genes at the genomic level.

### 3.5 Proper maintenance of myelinating Schwann cells depends on Dgcr8 and Dicer function

**Figure 5:**
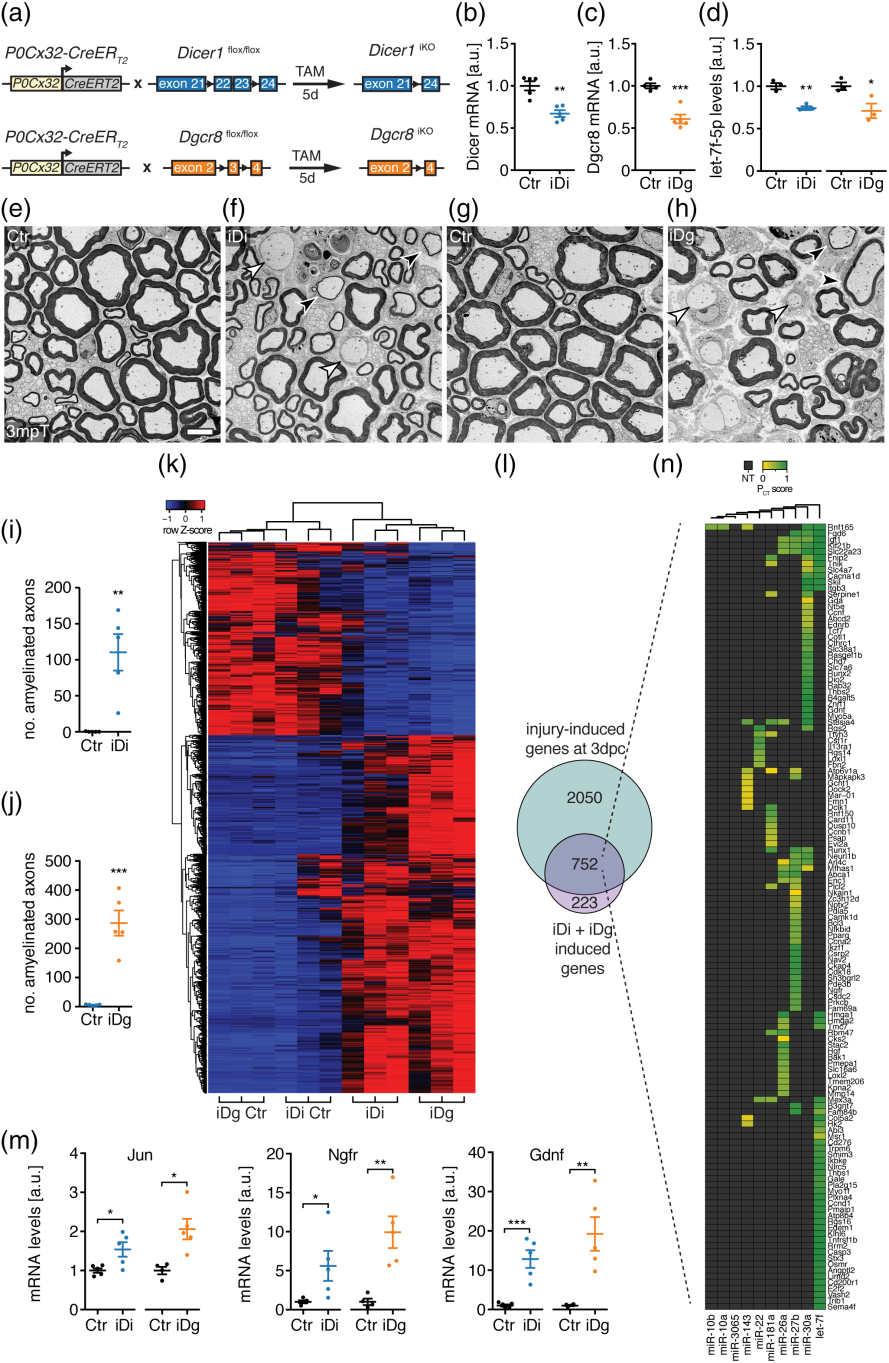
Dgcr8 and Dicer are required to properly maintain the myelinating SC fate. (a) Scheme of tamoxifen‐induced SC‐specific Dicer and Dgcr8 deletion strategy. (b,c) Levels of Dicer mRNA (b) in SNs of induced *P0‐Cx32* ^*CreERT2*^ *‐Dicer1* ^*flox/flox*^ (iDi) and Dgcr8 mRNA (c) in induced *P0‐Cx32* ^*CreERT2*^ *‐Dgcr8* ^*flox/flox*^ mice (iDg) compared to respective controls (Ctr) in 3 months post‐tamoxifen (mpT) SNs. Number (*n*) of mice per condition: Ctr (*n* = 4 and 5), iDi (*n* = 5), and iDg (*n* = 5). (d) Levels of let‐7f‐5p normalized to sno‐202 in 3 mpT SN of iDi and iDg compared to respective controls (Ctr) (*n* = 3 mice per condition). (e–h) Electron micrographs of iDi (f), iDg (h), and their respective controls (e,g). Examples of amyelinated (white arrowheads) and thinly myelinated axons (black arrowheads) are highlighted. (i,j) Number of amyelinated axons per SN cross‐section in iDi (i) and iDg (j) compared to their respective controls at 3 mpT (*n* = 5 mice per condition). (k) Heatmap of differentially expressed genes in iDi and iDg (fold change ≥2, FDR ≤ 0.05) compared to their respective controls (iDg Ctr and iDi Ctr) at 3 mpT. Color intensities represent row *Z*‐score, red indicating higher expression and blue indicating lower expression. (l) Venn diagram showing upregulated genes in 3 dpc SNs and in iDi and iDg (fold change ≥2, FDR ≤ 0.05). (m) Levels of Jun, Ngfr, and Gdnf mRNA in SNs of iDi and iDg compared to their respective controls at 3 mpT. Number (*n*) of mice per condition: Ctr (*n* = 4 and 5), iDi (*n* = 5), and iDg (*n* = 5). (*n*) Top10 miRNAs expressed at P60 and their predicted target sites (target scan) among the 752 genes induced in 3 dpc SNs and in iDg and iDi. Target site conservation and quality is represented by the P_CT_ score and nonpredicted targets (NT) in gray. Scale bar, 5 μm (e–h). All mutant animals used were preselected based on morphology (b–k,m). Error bars: *SEM.* Two‐sided two‐sample Student's *t* test *p < .05, **p < .01, ***p < .001 (b–d,i,j,m) [Color figure can be viewed at wileyonlinelibrary.com]

Given the de novo appearance of repair cell markers and the accumulation of injury‐related gene sets, we hypothesized that the miRNA biogenesis pathway might be even more generally necessary for the suppression of such gene programs in SCs. To address whether the miRNA pathway is required in this way also in adulthood, we employed a tamoxifen (TAM)‐inducible Cre recombinase system (CreERT2) driven by the 1.1 kb rat *Mpz* promoter fused to 5′‐untranslated parts of the human *GJB1* gene (Leone et al., 2003) allowing depletion of Dgcr8 (Dgcr8 iKO) and Dicer (Dicer iKO) specifically in adult myelinating SCs (Figure 5a). Morphological analysis at 3 months post‐TAM (mpT) revealed defects in myelin maintenance as indicated by amyelinated large‐caliber axons present in both Dicer and Dgcr8 iKO (Figure 5e–j). In addition, we observed axons with thin myelin sheaths in Dicer and Dgcr8 iKO indicative of on‐going remyelination (Figure 5f,h). However, not all DGCR8 and Dicer iKO animals were affected morphologically, in agreement with the incomplete penetrance previously reported in TAM‐induced Dgcr8 and Dicer mutants using inducible systems (Lin et al., 2015; Shin, Shin, McManus, Ptacek, & Fu, 2009; Viader et al., 2011). Assessment of toluidine blue‐stained sciatic nerve sections revealed 11 (out of 26) DGCR8 iKO animals without detectable changes between 3 and 6 mpT, and 4 (out of 11) Dicer iKO mice without detectable changes even at 14 mpT. Of note, incomplete penetrance was observed on both genders. As a consequence, we restricted the analysis to affected mice. As expected, levels of Dicer mRNA in Dicer iKO and Dgcr8 mRNA in Dgcr8 iKO were reduced in comparison to controls at 3 mpT (Figure 5b,c). In line with these findings, levels of let‐7f, an abundant miRNA in adult SNs, were reduced in Dicer and Dgcr8 iKO compared to controls (Figure 5d). To address whether the observed demyelination is accompanied by molecular signs of ongoing SC reprogramming, we performed whole‐transcriptome analysis of SNs of Dicer and Dgcr8 iKO at 3 mpT. Hierarchical cluster analysis of differentially expressed genes in Dgcr8 iKO revealed an overall similarity of the transcriptomes of Dicer and Dgcr8 iKO (Figure 5k), consistent with the observed morphological similarities. Furthermore, the majority (77%) of the jointly induced genes in Dicer and Dgcr8 iKO were also induced upon injury in 3 dpc SNs (Figure 5l), suggesting ongoing SC reprogramming. This was confirmed by detection of induced Jun, Ngfr, and Gdnf transcript levels in Dicer and Dgcr8 iKO compared to controls (Figure 5m). We also note that not all genes induced upon crush injury were also jointly induced in Dicer and Dgcr8 iKO (Figure 5l). Similarly, some of the jointly induced genes in Dicer and Dgcr8 iKO were not induced upon crush injury. This is expected and argues toward additional on‐going molecular and cellular processes that are not shared between crush injury and the myelin maintenance defects in Dicer and Dgcr8 iKO.

Next, we addressed whether abundant miRNAs in adult SNs are predicted to target the transcripts of the 752 genes jointly induced in 3 dpc, Dicer iKO, and Dgcr8 iKO. We found that 129 genes have predicted and conserved target sites within their 3′‐UTRs for the top 10 miRNAs expressed in P60 SNs. Similar to our predictions during development, these data support the idea that miRNAs are involved in the induction of the injury‐induced genes upregulated in Dicer and Dgcr8 iKO (Figure 5n). In summary, our data reveal a requirement of Dicer and Dgcr8 in proper maintenance of the myelinating SC fate, which we predict is likely due to a core set of SN‐enriched miRNAs targeting injury‐induced genes.

## 4 DISCUSSION

We have gathered data from a quantitative comparative loss‐of‐function approach targeting core components of the miRNA biogenesis pathway in SCs, combined with whole‐transcriptome and explorative bioinformatics analyses. Our results show the following: (a) The SC‐expressed microprocessor is essential for PNS development by guiding radial sorting of axons by SCs into myelinating units, ensuring correct SC numbers for this process. (b) The microprocessor is necessary to prevent aberrantly‐elevated transcript levels of injury‐response genes in developing SCs. (c) Bioinformatics analysis predicts that microprocessor‐dependent expression of injury‐response genes is targeted by developmental stage‐specific enriched miRNAs and differentially expressed TFs. (d) Dicer and the microprocessor component Dgcr8 are required for proper maintenance of the myelinating SC fate, presumably by maintaining miRNA‐mediated suppression of injury‐response genes.

Successful radial sorting depends on an accurate pool of immature SCs to match the number of axonal segments to be myelinated. The observed deficit in SCs in P1 Dgcr8 and Drosha cKO is therefore likely to be causal for the early impairments in radial sorting. Such impairments have also previously been observed in response to increased SC death or decreased SC proliferation (Brinkmann et al., 2008; Messing et al., 1992; Porrello et al., 2014). As proliferation was not detectably changed in Dgcr8 and Drosha cKO, the increase in SC apoptosis likely contributes to the observed SC deficit. Additionally, given the developmental induction of injury‐response genes in Dgcr8 and Drosha cKO, this may further lead to some form of early SC reprogramming, drifting away from the transcriptome boundaries normally required by immature SCs. In this way, such changes may also diminish the pool of immature SCs that are capable of performing radial sorting.

The remarkable ability of SCs to respond to injury and to activate a defined repair program is beneficial to the recovery of peripheral nerves and stands in sharp contrast to the CNS injury response (Brosius Lutz & Barres, 2014). However, such a program needs to be tightly controlled. Our results from developmental Dgcr8 and Drosha cKO reveal that the microprocessor is critically involved in this regulation of the injury‐response program by preventing its inappropriate activity during PNS development. In support of this interpretation, we detected prominent induction of Shh and Gdnf upon loss of microprocessor in P1 SN, both of which are described markers of repair SCs (Jessen & Mirsky, 2016). The presence of these markers at this time point coincides with the onset of the cellular phenotype observed upon loss of microprocessor. Along with the presence of these repair SC markers, we verified the expression of other injury‐response genes by comparing whole‐transcriptome data after loss of microprocessor at P1 with data gathered from 3 dpc SNs, consistent with an early reprogramming of microprocessor‐deficient SCs into repair‐like SCs. Nevertheless, microprocessor‐dependent genes that are not induced upon injury might also contribute to impaired radial sorting.

In adult myelinating SCs, we found that induced deletion of Dgcr8 or Dicer leads to a failure in proper maintenance of the myelinating SC fate, resulting in the presence of demyelinated axons. Additionally, both Dgcr8 and Dicer iKO express a large fraction of genes induced upon active SC reprogramming. Notably, the requirement of both Dgcr8 and Dicer to counteract injury‐response gene expression in adult SCs is in apparent contradiction to the requirement of the microprocessor, but not Dicer, in the suppression of these genes in developing SCs. However, the major relevant described functions of both the microprocessor and Dicer are in the biogenesis of canonical miRNAs. In this context, we show that stage‐specifically enriched miRNAs in developing and adult nerves are predicted to target a large fraction of the injury‐induced transcripts that are enriched upon loss of the microprocessor in developing SNs and upon induced Dgcr8 or Dicer deletion in adult SCs. Upon closer inspection, however, we noticed that the genes induced in developing SCs in Dgcr8 and Drosha cKO are also mildly increased in Dicer cKO. In the context of miRNAs‐based regulation, this might be interpreted in that miRNAs are less efficiently depleted and/or less functional in Dicer cKO than in Dgcr8 and Drosha cKO. Related to this, it has been reported that loss of Drosha compared to Dicer does not equally affect the population of expressed miRNAs (Kim, Kim, & Kim, 2016). While loss of Drosha caused a strong decrease of all expressed miRNAs, loss of Dicer more specifically resulted in reduced levels of miRNAs derived from the 3′‐strand (3p) leaving the 5′‐strand (5p) miRNAs less affected. The authors suggested a possible Dicer‐independent mechanism to mature pre‐miRNAs to mature 5p‐species. While such a mechanism might contribute to the milder radial sorting defects and the less prominent upregulation of the microprocessor‐dependent genes in Dicer cKO, differences in stabilities of the genetically targeted proteins‐to‐be‐deleted may also take part in shifting miRNA depletion efficiencies.

In Dicer and Dgcr8 iKO SN, we found incomplete phenotypic penetrance. Individual miRNA mutants often lack a phenotype or show incomplete penetrance if not challenged by additional environmental stress factors (Mendell & Olson, 2012). Thus, the lack of such stress factors together with desynchronized miRNA depletion timings in individual SCs may contribute to the penetrance variability that we observed.

Taken together, we favor a general mechanism involving miRNAs that prevents inappropriate activity of the injury response in developing and adult SCs. However, we cannot exclude other distinct mechanisms contributing to or accounting for the defects that we observed in our mutants during development and myelin maintenance. For example, the microprocessor was shown to bind also to pri‐miRNA‐like structures present in other RNA species such as mRNAs, small nuclear‐RNAs, and long non‐coding RNAs (Macias, Cordiner, & Caceres, 2013). During adult neurogenesis, Drosha, but not Dicer or miRNAs, is required to favor neuronal differentiation over oligodendrogenesis by binding and cutting a pri‐miRNA‐like hairpin within Nfib mRNA (Rolando et al., 2016). Similarly, for dendritic cell development, Drosha is required to cleave hairpins within mRNAs encoding inhibitors of myelopoiesis (Johanson et al., 2015).

We envision that our data, including the bioinformatics analysis, constitute an important resource that motivates multiple future studies to individually evaluate the physiological impact of predicted miRNA‐target and transcription factor relationships, which could act to safeguard early onset radial sorting and the myelinated adult SC state.

Our data highlight the multifaceted requirement of the miRNA biogenesis pathway in SC physiology during development and adulthood, potentially also with consequences in SC‐related pathologies as seen in neuropathies, or in the processes following after nerve injury and in regeneration. These aspects warrant further investigations.

## CONFLICT OF INTEREST

No conflict of interests declared.

## AUTHOR CONTRIBUTIONS

Conceptualization: DG, JAP, US; methodology: DG, JAP, LO, US; validation: DG, JAP, LO, US; formal analysis: DG, JAP, LO, AM; investigation: DG, JAP, DC, TK; resources: US; data curation: LO, DG; writing ‐ original draft: DG; writing ‐ review & editing: DG, JAP, LO, DC, TK, AM, US; visualization: DG, JAP; supervision: DG, JAP, US; project administration: US; funding acquisition: US.

## DATA AVAILABILITY

RNA sequencing data have been deposited at the NCBI GEO database with the primary accession code GSE109075.

## Supporting information


**Figure S1** Microprocessor‐independent miRNAs do not underlie the earlier developmental arrest of Dgcr8 cKO compared to Dicer. Related to Figure 1
**Figure S2**. Sciatic nerve cellularity and cytokine expression. Related to Figures 2 and 3
**Figure S3**. Gene set enrichment analysis of biological processes associated with differentially regulated genes after nerve crush injury. Related to Figure 4
**Table S1**. List of reagents
**Table S2**. List of qPCR primers

File S1 Predicted target genes and Pct scores of top 10 SN‐enriched miRNAs at P1.

File S2 Predicted target genes and Pct scores of top 10 SN‐enriched miRNAs at P60.

File S3 GO terms (biological process) for genes upregulated in Dgcr8 cKO and Drosha cKO at P1.

File S4 Transcription factors predicted to target differentially expressed genes in Dgcr8 cKO and Drosha cKO at P1.

File S5 GO terms (biological process) for genes upregulated upon SN injury in wild‐type mice.

File S6 GO terms (biological process) for genes downregulated upon SN injury in wild‐type mice.

File S7 Transcription factors predicted to target differentially expressed genes upon SN injury in wild‐type mice.
