# Supplementary material for: The miRNA biogenesis pathway prevents inappropriate expression of injury response genes in developing and adult Schwann cells
Source: Glia. 2018 Oct 8;66(12):2632–44. doi: 10.1002/glia.23516 (PMC6585637; doi:10.1002/glia.23516)
Supplement: Supplementary file 1 — Figure S1 Microprocessor‐independent miRNAs do not underlie the earlier developmental arrest of Dgcr8 cKO compared to Dicer. Related to Figure 1 Figure S2. Sciatic nerve cellularity and cytokine expression. Related to Figures 2 and 3 Figure S3. Gene set enrichment analysis of biological processes associated with differentially regulated genes after nerve crush injury. Related to Figure 4 Table S1. List of reagents Table S2. List of qPCR primers [file GLIA-66-2632-s001.docx]

**Supporting Information**


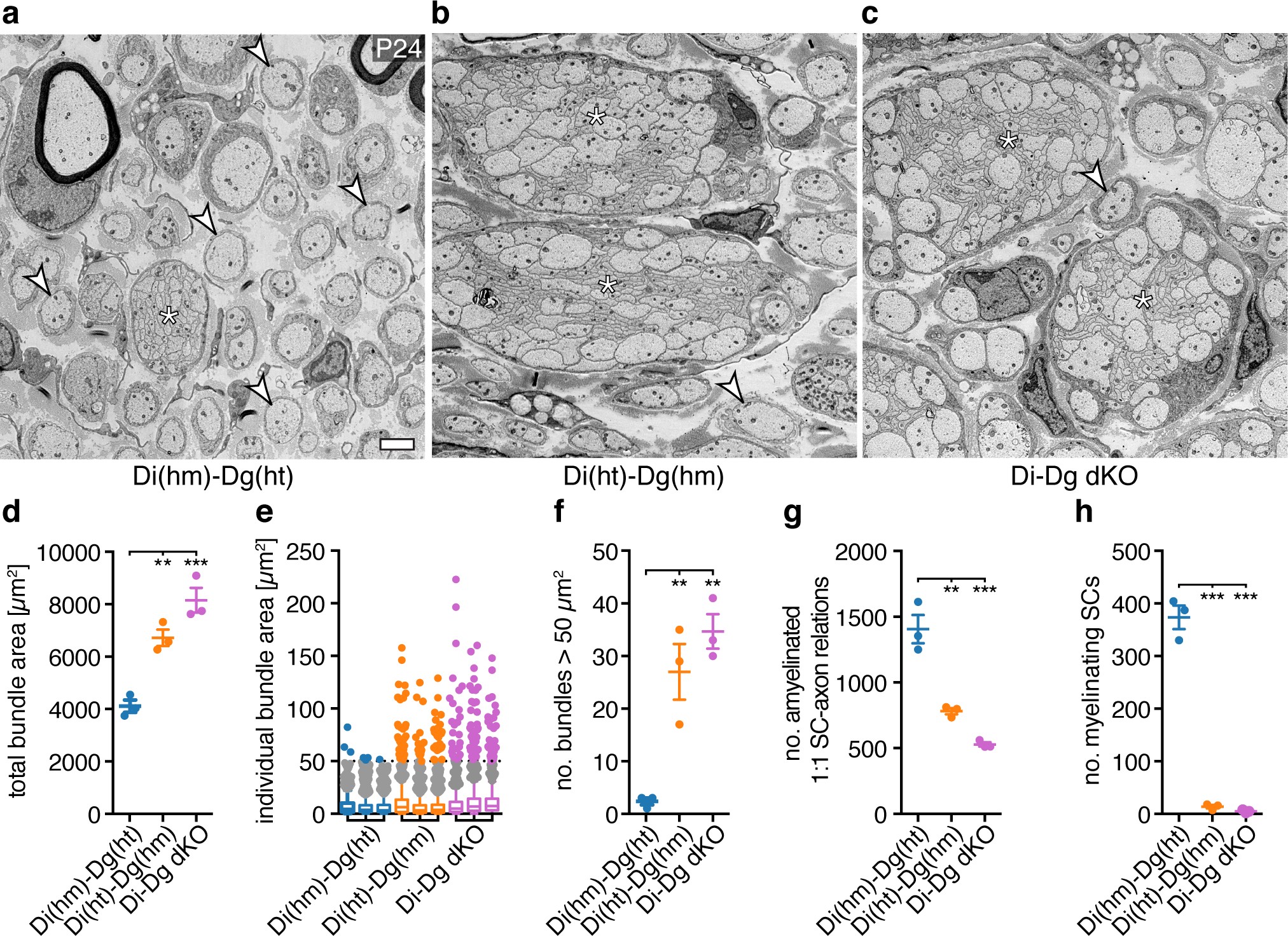


**Figure S1.** Microprocessor-independent miRNAs do not underlie the earlier developmental arrest of Dgcr8 compared to Dicer cKO. Related to Figure 1. (a-c) Electron micrographs of *Dhh^Cre^-Dicer1^flox/flox^-Dgcr8^flox/wt^* (Di(hm)-Dg(ht)) (a), *Dhh^Cre^-Dicer1^flox/wt^-Dgcr8^flox/flox^* (Di(ht)-Dg(hm)) (b), and *Dhh^Cre^-Dicer1^flox/flox^- Dgcr8^flox/flox^* mutant mice (Di-Dg dKO) (c). (d) Total area covered by bundles of unsorted axons per SN cross-section in Di(hm)-Dg(ht), Di(ht)-Dg(hm) and Di-Dg dKO at P24 (3 mice per condition). (e) Box plots representing individual bundle size distribution per SN cross-section of Di(hm)-Dg(ht), Di(ht)-Dg(hm) and Di-Dg dKO at P24 (one box per animal). (f) Number of bundles of unsorted axons larger than 50 µm^2^ in SNs of Di(hm)-Dg(ht), Di(ht)-Dg(hm) and Di-Dg dKO at P24 (3 mice per condition). (g,h) Number of 1:1 amyelinated SC-axon units (g) and myelinating SCs (h) per SN cross-section in Di(hm)-Dg(ht), Di(ht)-Dg(hm) and Di- Dg dKO at P24 (3 mice per condition). Scale bar, 2 µm (a-c). Error bars: s.e.m. One-way ANOVA with Tukey’s multiple comparison test **P*<0.05, ** *P*<0.01, *** *P*<0.001 (d, f-h).


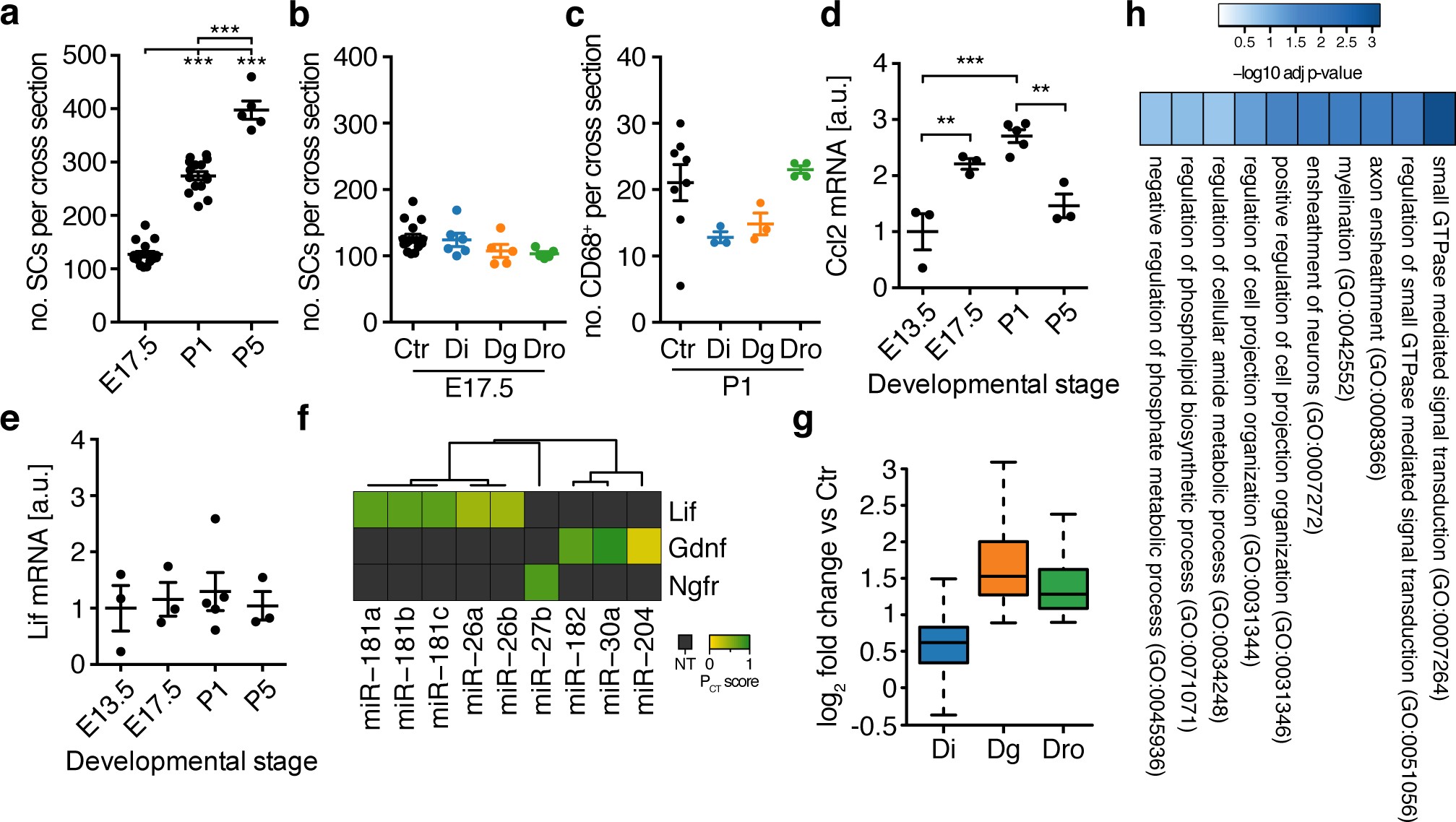


**Figure S2.** Sciatic nerve cellularity and cytokine expression. Related to Figure 2 and 3. (a) Number of SCs per SN cross-section in control mice at E17.5, P1 and P5. Number (n) of mice per condition: E17.5 (n=16), P1 (n=15), P5 (n=5). E17.5 control data are also represented in Figure S2B, and P1 control data are also represented in Figure 2B. (b) Number of SCs per SN cross-section in Dicer cKO (Di), Dgcr8 cKO (Dg) and Drosha cKO (Dro) compared to controls (Ctr) at E17.5. Number (n) of mice per condition: Ctr (n=16), Di (n=6), Dg (n=5), Dro (n=5). (c) Number of CD68-positive cells per SN cross-section in Ctr, Di, Dg and Dro compared to Ctr at P1. Number (n) of mice per condition: Ctr (n=8), Di (n=3), Dg (n=3), Dro (n=4). (d,e) Levels of Ccl2 (d) and Lif (e) mRNA in peripheral nerves at E13.5, E17.5, P1 and P5. Number (n) of mice per condition: E13.5 (n=3), E17.5 (n=3), P1 (n=5), P5 (n=3). (f) Predicted miRNA target sites (TargetScan) within Lif, Gdnf and Ngfr 3’-UTRs among Top50-expressed miRNAs (Gökbuget et al., 2015) in P1 SN (TargetScan). Target site conservation and quality is reflected by the P_CT_ score and non-predicted targets (NT) in gray. Error bars: s.e.m. One-way ANOVA with Tukey’s multiple comparison test **P*<0.05, ** *P*<0.01, *** *P*<0.001 (a-e). (g) Log_2_-fold change of Dg and Dro upregulated genes (Figure 3c) in Di, Dg and Dro compared to controls. (h) GO analyses of downregulated genes in Dgcr8 and Drosha cKO compared to Dicer cKO and controls.


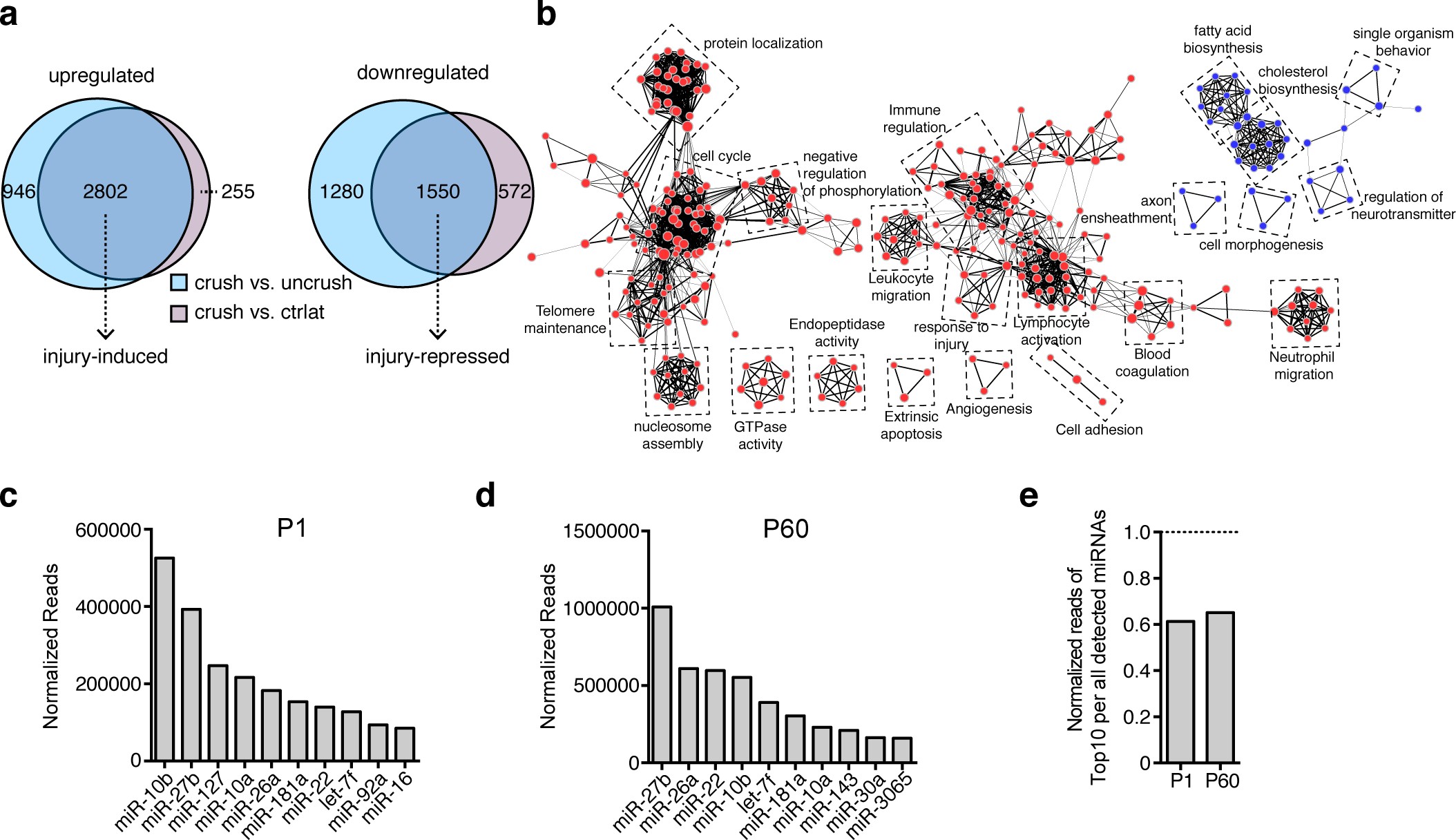


**Figure S3.** Gene set enrichment analysis of biological processes associated with differentially regulated genes after nerve crush injury. Related to Figure 4.

1. Venn diagram comparing number of up- and downregulated genes in RNA sequencing data from 3 dpc SNs (crush) compared to uncrushed and contralateral (ctrlat) controls. Number (n) of mice per condition: crush (n=4), ctrlat (n=4), uncrushed (n=3). Jointly up- and down-regulated genes were considered as injury-induced and –repressed genes in downstream bioinformatics analyses.
2. Network graph produced by Cytoscape 3 showing significantly enriched GO biological process gene sets among the injury-induced (red nodes) and - repressed (blue nodes) genes imported into Enrichment Map. Nodes represent individual GO term gene sets. Edges represent the relatedness between the genes within the gene sets. (c,d) Normalized reads (by library size) of Top10 miRNAs expressed in sciatic nerve extracts at P1 (c) and P60 (d) (Gökbuget et al., 2015). (e) Relative abundance of Top10 miRNAs compared to all other detected miRNAs in SN extracts of P1 and P60.

**Table S1.** List of reagents.

| REAGENT | SOURCE | IDENTIFIER |
| --- | --- | --- |
| Antibodies | | |
| Rabbit-anti-Cleaved Caspase 3 | Cell Signaling | 9661 |
| Goat-anti-Sox10 | R&D | AF2864 |
| Rat-anti-CD68 | Serotec | MCA1957 |
| Donkey-anti-rabbit-Alexa488 | Life Technologies | A21206 |
| Donkey-anti-goat-Alexa 647 | Life Technologies | A21447 |
| Donkey-anti-rat-Alexa 647 | Life Technologies | A21247 |
| Commercial assays, chemicals and enzymes | | |
| TruSeq Stranded total RNA Sample Prep Kit | Illumina | 20020596 |
| RiboZero Gold rRNA Removal Kit | Illumina | MRZG126 |
| Fast Start Essential DNA Green Master | Roche | 06924204001 |
| TaqMan Universal PCR Master Mix | Life Technologies | 4324018 |
| TaqMan MicroRNA Assay let-7f-5p | Life Technologies | 000382 |
| TaqMan MicroRNA Assay miR-10a-5p | Life Technologies | 000387 |
| TaqMan MicroRNA Assay miR-181a-5p | Life Technologies | 000480 |
| TaqMan MicroRNA Assay miR-30a-5p | Life Technologies | 000417 |
| DAPI | Sigma | D9542 |
| Click-iT EdU Alexa Fluor™ 647 Imaging Kit | Invitrogen | C10340 |
| EdU (5-ethynyl-2’-deoxyuridine) | Invitrogen | E10187 |
| TUNEL enzyme | Roche | 11767305001 |
| Biotin-16-dUTP | Roche | 11093070910 |
| Streptavidin Alexa Fluor 546 | Invitrogen | S11225 |
| Software and Algorithms | | |
| Trimmomatic | (Bolger et al., 2014) | Version 0.33 |
| STAR | (Dobin et al., 2013) | Version 2.5.1b |
| RSEM | (Li and Dewey, 2011) | Version 1.2.22 |
| edgeR | (Robinson et al., 2010) | Version 3.12 |
| Biolayout Express3D | <http://www.biolayout.org/> | Version 3.2 |

**Table S2.** List of qPCR primers.

**Primer Sequence (5’-3’)**

dicer1_forward GGCTTCCTCCTGGTTATGTGGTAAACC dicer1_reverse ACCCAGTTTGCCATTAGCCAGC dgcr8_forward CTGTGCTCCCAAGAAGAGGCG dgcr8_reverse CAATGGCTCTGTAGGTGGACGG drosha_forward GACGACGACAGCACCTGTT

drosha_reverse GATAAATGCTGTGGCGGATT shh_forward AAAGCTGACCCCTTTAGCCTA shh_reverse TTCGGAGTTTCTTGTGATCTTCC gdnf_forward CGCTGACCAGTGACTCCAAT gdnf_reverse GCTGCCGCTTGTTTATCTGG

ngfr_forward GGGCCTTGTGGCCTATATTGCTTTCAAGAG

ngfr_reverse CTGTCGCTGTGCAGTTTCTCTCCCTC jun_forward CCTTCTACGACGATGCCCTC jun_reverse GGTTCAAGGTCATGCTCTGTTT ccl2_forward AGGTCCCTGTCATGCTTCTG ccl2_reverse GCTGCTGGTGATCCTCTTGT plau_forward AGGGGGAGCACTGTGAGATA plau_reverse AGGTCTGTGGGCATTGTAGG lif_forward ATTGTGCCCTTACTGCTGCTG lif_reverse GCCAGTTGATTCTTGATCTGGT timp1_forward GCAACTCGGACCTGGTCATAA timp1_reverse CGGCCCGTGATGAGAAACT actb_forward TTCTTTGCAGCTCCTTCGTT actb_reverse ATGGAGGGGAATACAGCCC

**File S1.** Predicted target genes and P_ct_ scores of top 10 SN-enriched miRNAs at P1.

**File S2.** Predicted target genes and P_ct_ scores of top 10 SN-enriched miRNAs at P60.

**File S3.** GO terms (biological process) for genes upregulated in Dgcr8 cKO and Drosha cKO at P1.

**File S4.** Transcription factors predicted to target differentially expressed genes in Dgcr8 cKO and Drosha cKO at P1.

**File S5.** GO terms (biological process) for genes upregulated upon SN injury in wild-type mice.

**File S6.** GO terms (biological process) for genes downregulated upon SN injury in wild-type mice.

**File S7.** Transcription factors predicted to target differentially expressed genes upon SN injury in wild-type mice.
